# Old drugs with new skills: fenoprofen as an allosteric enhancer at melanocortin receptor 3

**DOI:** 10.1007/s00018-016-2419-3

**Published:** 2016-11-16

**Authors:** Trinidad Montero-Melendez, Rachel A. E. Forfar, Jennifer M. Cook, Jeffrey C. Jerman, Debra L. Taylor, Mauro Perretti

**Affiliations:** 1 The William Harvey Research Institute, Barts and The London School of Medicine, Queen Mary University of London, Charterhouse Square, London, EC1M 6BQ UK; 2 Medical Research Council Technology, Centre for Therapeutics Discovery, 1-3 Burtonhole Lane, Mill Hill, London, NW7 1AD UK

**Keywords:** Inflammation, Arthritis, GPCR, Drug repositioning, Resolution of inflammation

## Abstract

The efficiency of drug research and development has paradoxically declined over the last decades despite major scientific and technological advances, promoting new cost-effective strategies such as drug repositioning by systematic screening for new actions of known drugs. Here, we performed a screening for positive allosteric modulators (PAMs) at melanocortin (MC) receptors. The non-steroidal anti-inflammatory drug fenoprofen, but not the similar compound ibuprofen, presented PAM activity at MC_3_, MC_4_, and MC_5_ receptors. In a model of inflammatory arthritis, fenoprofen afforded potent inhibition while ibuprofen was nearly inactive. Fenoprofen presented anti-arthritic actions on cartilage integrity and synovitis, effects markedly attenuated in Mc3r−/− mice. Fenoprofen displayed pro-resolving properties promoting macrophage phagocytosis and efferocytosis, independently of cyclooxygenase inhibition. In conclusion, combining repositioning with advances in G-protein coupled receptor biology (*allosterism*) may lead to potential new therapeutics. In addition, MC_3_ PAMs emerged as a viable approach to the development of innovative therapeutics for joint diseases.

**Electronic supplementary material:**

The online version of this article (doi:10.1007/s00018-016-2419-3) contains supplementary material, which is available to authorized users.

## Introduction

In recent years, we have witnessed a steady and progressive decline in productivity of the pharmaceutical industry despite increased investments in R&D, leading to absence of medicines active against the diseases of modern societies [38]. To obviate to this financial and medical problem, drug repositioning has emerged as a new business model to reduce developing timeframe, costs, and improve success rates. Identification of new indications for old drugs (typically generics or drugs that failed approval for reasons others than safety) has proven to be a feasible approach as demonstrated, for instance, by the recent successful repositioning of thalidomide and sildenafil [31]. Compound libraries containing approved drugs offer an opportunity for systematic screening for potential new uses of existing drugs, while simultaneously identifying novel candidates, as it is accepted that drug repositioning by itself will not be sustainable as sole development model for the medicines required by western societies. However, merely by including known molecules in drug screenings might not represent a substantial improvement in success rate as the complexity of disease mechanisms and still limited understanding of whole biological systems (compared to one-cell-at-a-time assays) are behind the unexpected lack of efficacy of promising drugs when tested in phase II and III human trials.

The field of G-protein coupled receptors (GPCR), considered one of the largest classes for drug discovery, has experienced a significant progress in recent years leading to innovative approaches for the pharmacological exploitation of these receptors including identification of biased ligands and allosteric modulators. One of these promising targets in the area of inflammation research is the melanocortin (MC) receptors. Pharmacological strategies for targeting the MC system are emerging as viable therapeutic approaches for a number of diseases, including obesity, cachexia, vitiligo, sexual dysfunction, dermatitis, and gouty arthritis [26]. Establishing receptor predominance in a given condition, out of the five receptors identified (MC_1_–MC_5_), together with development of selective agonists, is of paramount importance to avoid off-target side effects. However, two decades of extensive search for selective molecules using classical orthosteric ligand approaches did not deliver any successful drug. Contrasting with this lack of lead compounds, there is a renewed interest in targeting the MC system derived from the appreciation of a non-cortisol-mediated properties of the MC peptide ACTH which involve activation of peripheral MC receptors, including MC_3_ [12, 26]. Activation of MC_3_ by its endogenous ligands, ACTH and melanocyte-stimulating hormone (MSH), attenuates inflammatory joint diseases in models mimicking rheumatoid arthritis (RA) and gout. Treatment of arthritic mice with the synthetic peptides DTrp^8^-γMSH and AP214 reduces disease severity in the K/BxN serum transfer model [29, 30, 34], while the natural peptides αMSH, γ_2_MSH, and MT-II (a stable derivative of αMSH) inhibit urate crystal inflammation [4, 11]. Notwithstanding this, the difficulty in achieving selective agonism has been hampering full harnessing of MC biology for innovative drug discovery programmes until now.

A promising strategy to attain selective activation of closely related G-protein coupled receptors (GPCR) is based on the development of allosteric ligands [1, 25, 33]. In contrast to orthosteric sites, where the natural ligands bind, allosteric sites (i.e., sites spatially distinct from endogenous ligands binding sites) display a higher degree of sequence divergence, providing new opportunities for the identification of selective compounds [9, 42]. Positive allosteric modulators (PAMs) or allosteric enhancers, potentiate receptor signaling in the presence of the endogenous ligand thus maintaining both temporal and spatial control, and may be burdened by fewer side effects in view of their likely higher degree of selectivity [9, 42]. Recently, Pantel et al. performed a screening on potential PAMs at MC_4_ to identify novel drugs for obesity with reduced side-effects profile, yielding 62 positive modulators [33].

We screened, here, a library of known small molecules for PAM activity at MC_3_, identifying the marketed drug fenoprofen (Fenopron^®^, UK, Nalfon^®^, US) as a positive hit. Long known for its anti-arthritic activities and efficacy in RA and osteoarthritis (OA) [17] fenoprofen is a non-steroidal anti-inflammatory drug (NSAID) and inhibits cyclooxygenase (COX) but as stated in the label, “its exact mode of action is unknown”. Here, we identify a new skill of fenoprofen (fundamentally distinct from its known COX-related mode of action) that may lead to further functional studies as a unique tool compound, and perhaps reconsider its use in conditions where the MC system has a substantial role. In addition, our study reveals an important contribution of MC_3_ in the therapeutic actions of fenoprofen in arthritis, providing proof of concept support to the allosteric enhancing mode of targeting the MC system for the treatment of inflammatory diseases.

## Results

### Fenoprofen is a positive allosteric modulator at the MC_3_ receptor

**Fig. 1:**
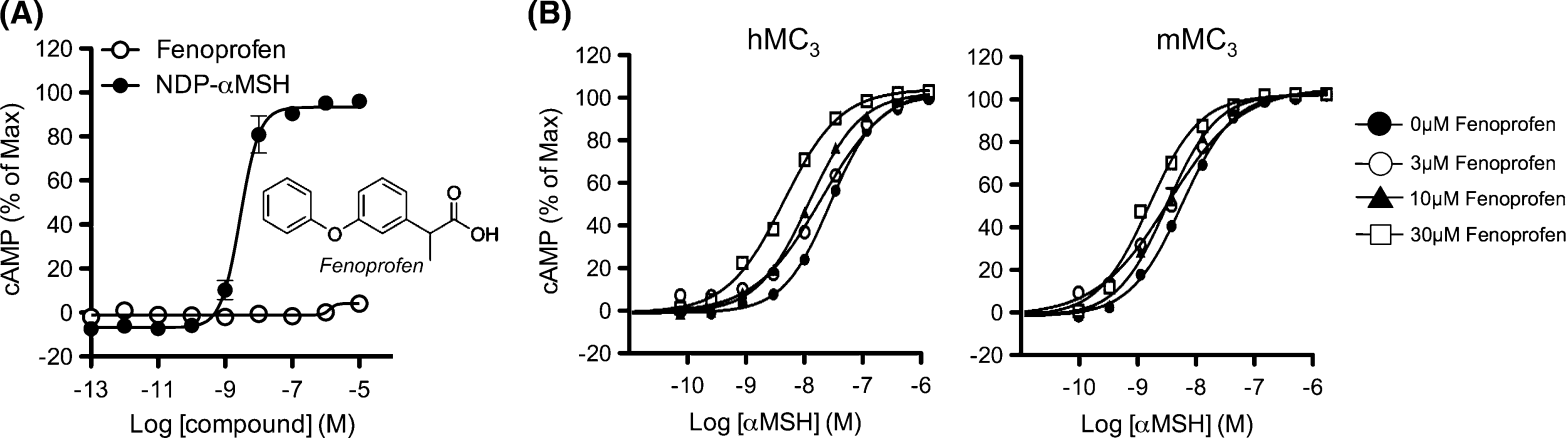
Allosteric modulatory activity of fenoprofen at the human MC_3_ receptor. cAMP production was studied in human MC_3_ transfected CHO-K1 cells (GeneBLAzer^®^ beta-lactamase Reporter Technology). Fenoprofen was tested for agonistic activity (**a**, agonist mode) or in the presence of the agonist αMSH to address PAM activity in human and mouse receptors (**b**, PAM mode)

Screening on a commercial library of known pharmacologically active molecules (>1400 compounds) using CHO cells stably expressing MC_3_ was run, using both an “agonist mode” (cAMP production) and a “PAM mode” (enhancement of cAMP production in the presence of a suboptimal (~EC_20_) concentration of αMSH). Among the most potent positive hits, the marketed NSAID fenoprofen emerged as a PAM at human MC_3_. Fenoprofen did not induce cAMP production when tested alone (Fig. 1a) but induced a leftward-shift of the αMSH concentration response in both human and mouse receptors (Fig. 1b). Fenoprofen was not agonist at other MCRs (Supplementary Figure S1) but displayed PAM activity at human MC_3_, MC_4_, and MC_5_ as well as mouse MC_3_, when αMSH, ACTH, and Lys-γ_3_MSH were used as endogenous ligands (Table 1), indicating that the PAM activity of fenoprofen was in general terms non-receptor and non-probe selective.

**Table 1:** Positive allosteric modulator activity of fenoprofen at melanocortin receptors

Drug	Receptor							
	mMC_3_		hMC_3_^(^*^)^		hMC_4_		hMC_5_	
NDP-αMSH	pEC_50_	Log shift	pEC_50_	Log shift	pEC_50_	Log shift	pEC_50_	Log shift
+0 μM Fen	9.78		9.40		10.49		8.98	
+3 μM Fen	9.86	0.08	9.51	0.11	–		8.97	−0.01
+10 μM Fen	9.76	−0.02	9.58	0.19	10.47	−0.02	9.06	0.08
+30 μM Fen	9.71	−0.07	9.66	0.26	10.38	−0.11	8.99	0.01

Drug	Receptor							
	mMC_3_^(^*^)^		hMC_3_^(^*^)^		hMC_4_^(^*^)^		hMC_5_^(^*^)^	
αMSH	pEC_50_	Log shift	pEC_50_	Log shift	pEC_50_	Log shift	pEC_50_	Log shift
+0 μM Fen	8.33		7.58		8.38		6.64	
+3 μM Fen	8.42	0.10	7.67	0.09	–		6.75	0.11
+10 μM Fen	8.58	0.25	7.92	0.34	8.80	0.41	6.89	0.25
+30 μM Fen	8.86	0.53	8.30	0.71	9.11	0.73	7.22	0.58

Drug	Receptor							
	mMC_3_^(^*^)^		hMC_3_^(^*^)^		hMC_4_^(^*^)^		hMC_5_^(^*^)^	
ACTH	pEC_50_	Log shift	pEC_50_	Log shift	pEC_50_	Log shift	pEC_50_	Log shift
+0 μM Fen	8.69		8.19		8.76		6.78	
+3 μM Fen	8.72	0.04	8.29	0.10	–		6.84	0.06
+10 μM Fen	8.85	0.16	8.57	0.38	9.09	0.33	7.03	0.25
+30 μM Fen	9.03	0.34	8.87	0.68	9.25	0.48	7.29	0.51

Drug	Receptor							
	mMC_3_		hMC_3_^(^*^)^		hMC_4_^(^*^)^		hMC_5_^(^*^)^	
Lys-γ_3_MSH	pEC_50_	Log shift	pEC_50_	Log shift	pEC_50_	Log shift	pEC_50_	Log shift
+0 μM Fen	9.19		8.80		7.33		6.13	
+3 μM Fen	9.24	0.05	8.89	0.09	–		6.31	0.18
+10 μM Fen	9.30	0.11	9.09	0.29	7.48	0.14	6.61	0.48
+30 μM Fen	9.27	0.08	9.35	0.55	7.72	0.39	6.80	0.67

Leftward log shift of the peptide ligand and EC_50_ values are reported at murine MC_3_ and human MC_3,4,5_ receptors (*indicates PAM activity)

Next, we addressed if MC receptor PAM activity was a common feature of other COX inhibitors. We focused on the NSAID family of propionic acid derivatives to which fenoprofen belongs [17]. Of the seven COX inhibitors tested, including ibuprofen, none potentiated melanocortin-induced cAMP production (Supplementary Table S1 and Supplementary Figure S2), indicating that the structural and physicochemical requirements for COX inhibition do not translate to MC_3_ PAM activity, with fenoprofen being unique in its class for this bioaction.

### MC_3_ mediates the anti-arthritic actions of fenoprofen

**Fig. 2:**
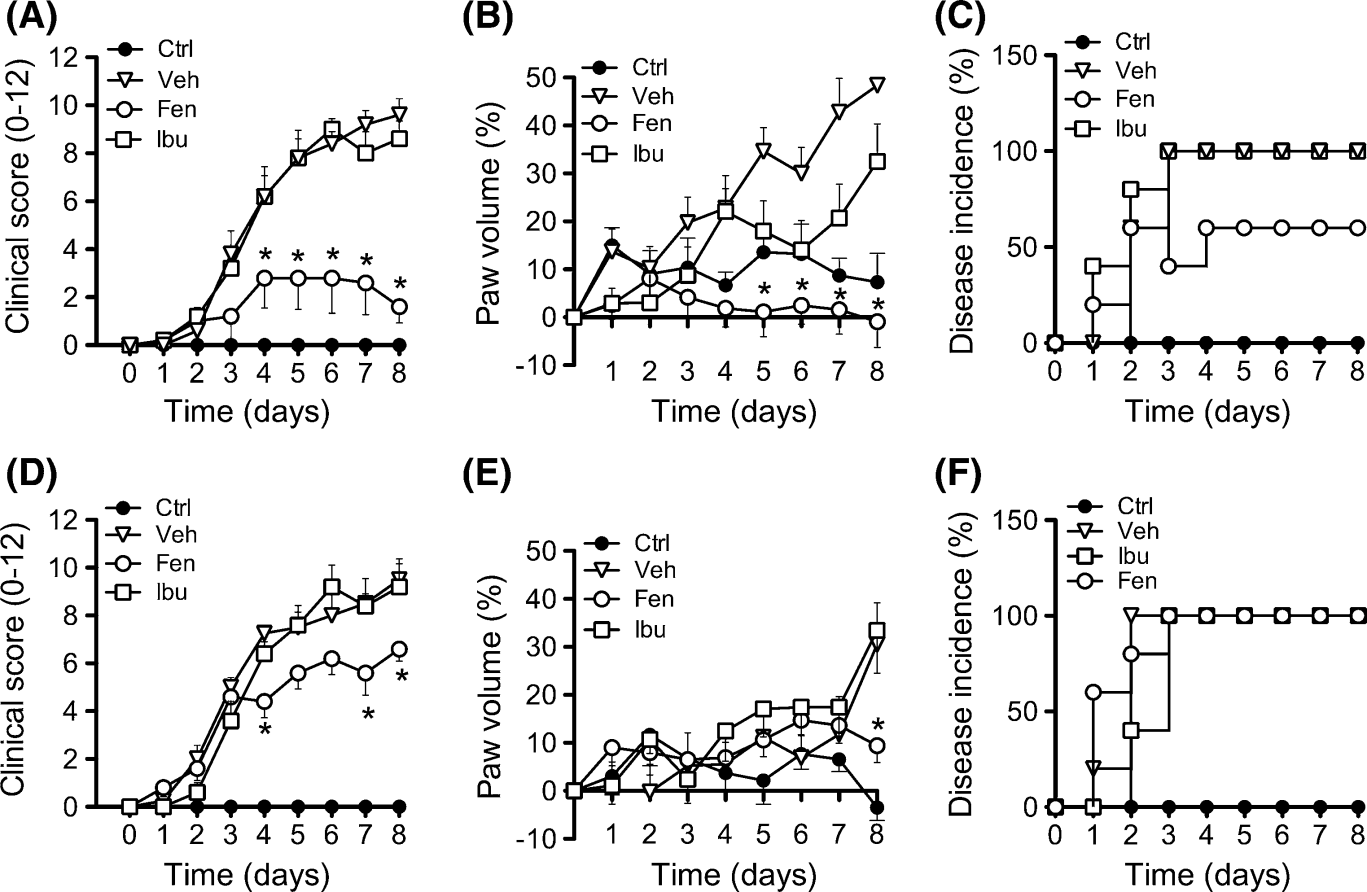
Antiarthritic effect of fenoprofen in the K/BxN serum transfer model. Arthritis was induced on wild type (**a**–**c**) and Mc3r−/− (**d**–**f**) mice by two i.p. injections of arthritogenic serum on day 0 and 2. Pharmacological treatments (fenoprofen, Fen, and ibuprofen, Ibu: 10 mg/kg; vehicle: PBS) were administered i.p. twice daily from day 2. Non-arthritic mice were used as controls (Ctrl). Clinical score (**a**, **d**), paw volume (**b**,**e**) and disease incidence (**c**,**f**) were recorded over 8 days. Data are mean ± SEM of *n* = 5; *p < 0.05 ANOVA followed by Bonferroni multiple comparison test

Since MC_3_ is emerging as a crucial receptor in controlling the severity of inflammatory arthritis, we next studied its potential involvement in the anti-arthritic actions of fenoprofen. The K/BxN serum transfer model of arthritis was induced in both wild type (WT) and MC_3_ deficient mice (Mc3r−/−). Arthritis developed normally in both strains acquiring moderate to severe disease at days 4–5 (Fig. 2). Pharmacological treatment of mice with fenoprofen or ibuprofen (10 mg/kg twice per day from day 2; dose selected from a pilot peritonitis—see Supplementary Figure S3) revealed distinct modulation of the disease. While fenoprofen had a significant impact on clinical score, with 80% reduction at day 8 (Fig. 2a) and paw volume (~100% reduction; Fig. 2b), ibuprofen was much less effective affording only a moderate response on edema with no effect on clinical score. Fenoprofen, but not ibuprofen, also reduced disease incidence (Fig. 2c). A large majority of the inhibitory properties of fenoprofen was lost in Mc3r−/− mice (Fig. 2d, e), with residual effectiveness of ~30% both for clinical score and paw volume. All Mc3r−/− mice treated with fenoprofen developed arthritis (Fig. 2f). Identical dosing with Ibuprofen was not particularly effective in this model, resulting in effects ranging from 5 to 30% inhibition for the majority of parameters tested. Therefore, absence of Mc3r brings fenoprofen efficacy comparable to that evoked by ibuprofen.

**Fig. 3:**
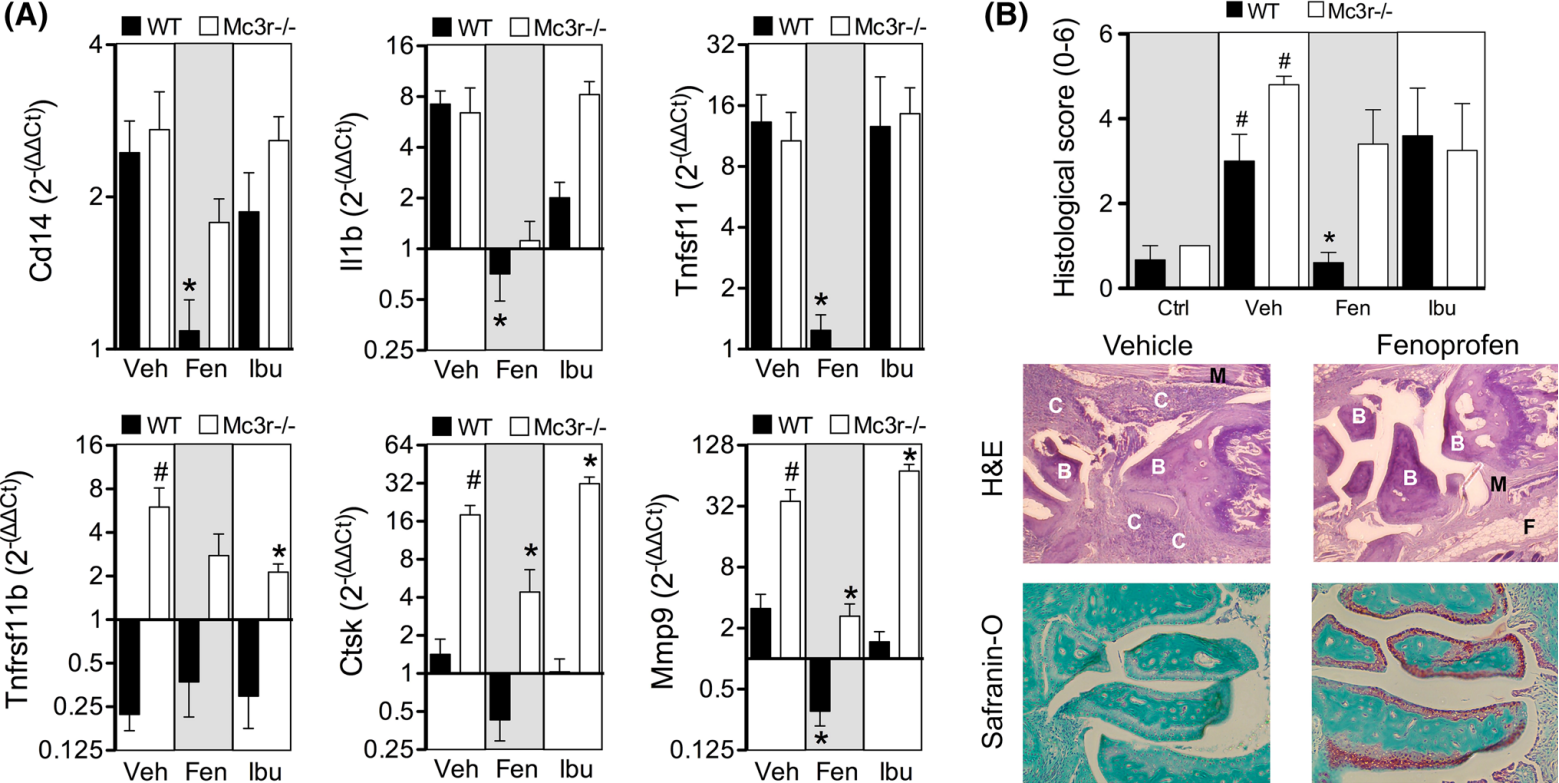
Gene expression and histological changes in arthritic joints. **a** Gene expression was analyzed in ankles from wild type (WT, *black bars*) and Mc3r−/− (*white bars*) mice as collected at day 8, and expressed as fold change with respect to non-arthritic control mouse joints. Groups correspond to vehicle (Veh), fenoprofen 10 mg/kg (Fen), and ibuprofen 10 mg/kg (Ibu). Data are mean ± SEM of *n* = 5; *p < 0.05 two-way ANOVA followed by Bonferroni multiple comparison test of veh vs. drugs, ^#^ *p* < 0.05 WT vs. Mc3r−/−. **b** Ankle sections (4 µm) were stained with hematoxylin and eosin (H&E) and fast green and safranin-O. Sections were graded from 0 (no disease) to 3 (severe) based on the degree of synovitis (purple staining in the H&E sections) and cartilage erosion (loss of *red coloration* in the safranin-O sections). *C* cell infiltrate, *B* bone, *M* muscle, *F* fat. The sum of H&E and safranin-O scores are represented in the *left graph*. Data are mean ± SEM of *n* = 5; *p* < 0.05 drug vs. Veh (*), or Veh vs. Ctrl (^#^) ANOVA followed by Bonferroni multiple comparison test

Gene expression analyses demonstrated a selective anti-inflammatory action of fenoprofen with marked reduction on monocyte marker Cd14, interleukin 1β (Il1b), RANK ligand (Tnfsf11), cathepsin K (Ctsk), and metalloproteinase 9 (Mmp9) (Fig. 3a). Ibuprofen mildly reduced Il1b expression albeit not significantly. Consistent with the known effect of MC_3_ agonism on cell recruitment [14, 30], the effect on Cd14 was absent in Mc3r−/− mice suggesting that endogenous MC_3_ mediated this particular action of fenoprofen. Interestingly, elevated levels of osteoprotegerin (Tnfrsf11b), Ctsk, and Mmp9 in Mc3r−/− vehicle-treated mice compared to WT suggest a defective bone metabolism in mice lacking this receptor. Histological analyses accounting for cell infiltration (H&E) and cartilage integrity (safranin-O) (see Supplementary Figure S4 for scoring criteria) reiterated the potent inhibitory actions of fenoprofen in the joints of WT but not in Mc3r−/− mice (Fig. 3b). Ibuprofen did not exert tissue protective actions.

These results indicate an important contribution of MC_3_ to the anti-arthritic actions of fenoprofen in vivo.

### Local increase of endogenous melanocortin peptides at inflammatory sites

**Fig. 4:**
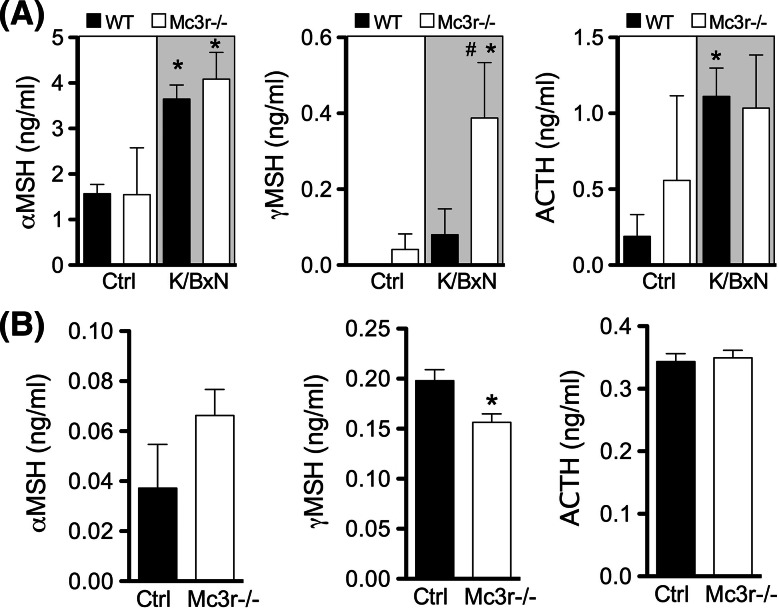
Production of endogenous melanocortin peptides in arthritic mouse joints and primary macrophages. **a** Wrists joints from K/BxN arthritic mice (wild type and Mc3r−/−) were collected at day 8 and protein extracts prepared on RIPA buffer. The melanocortin peptides αMSH, γMSH, and ACTH were determined by enzyme immunoassay following manufacturers’ protocol. **b** Biogel-elicited macrophages were collected from mice (wild type and Mc3r−/−) 4 days after an i.p. injection with 1 ml of 2% biogel. Cells were cultured in vitro during 24 h in 10% FCS-RPMI. Supernatants were analyzed for αMSH, γMSH, and ACTH levels by enzyme immunoassay. Data are mean ± SEM of *n* = 5; *p* < 0.05 ANOVA followed by Bonferroni multiple comparison test K/BxN vs. Ctrl (*) or WT vs. Mc3r−/− (^#^)

Allosteric enhancers exert their biological effects only in the presence of the endogenous agonist. Thus, we measured levels of melanocortin peptides in arthritic (day 8) and naïve joints. Interestingly, αMSH and ACTH were significantly increased in the inflamed joints of arthritic mice compared to non-arthritic mice (Fig. 4a), while plasma levels remained stable with values within the 3–5 ng/ml range (Supplementary Figure S5). γMSH levels were very low or not detectable in control joints. The elevated levels of melanocortin peptides measured in arthritic joints provide mechanistic support to the PAM efficacy of fenoprofen. Moreover, measurements in Mc3r−/− tissues demonstrated elevated values for γMSH suggesting (1) the existence of a negative loop between receptor expression and its high-affinity natural ligand and (2) that the lack of effect of fenoprofen in this genotype was not secondary to inadequate production of the endogenous ligands.

### Fenoprofen exerts pro-resolving actions via MC_3_

**Fig. 5:**
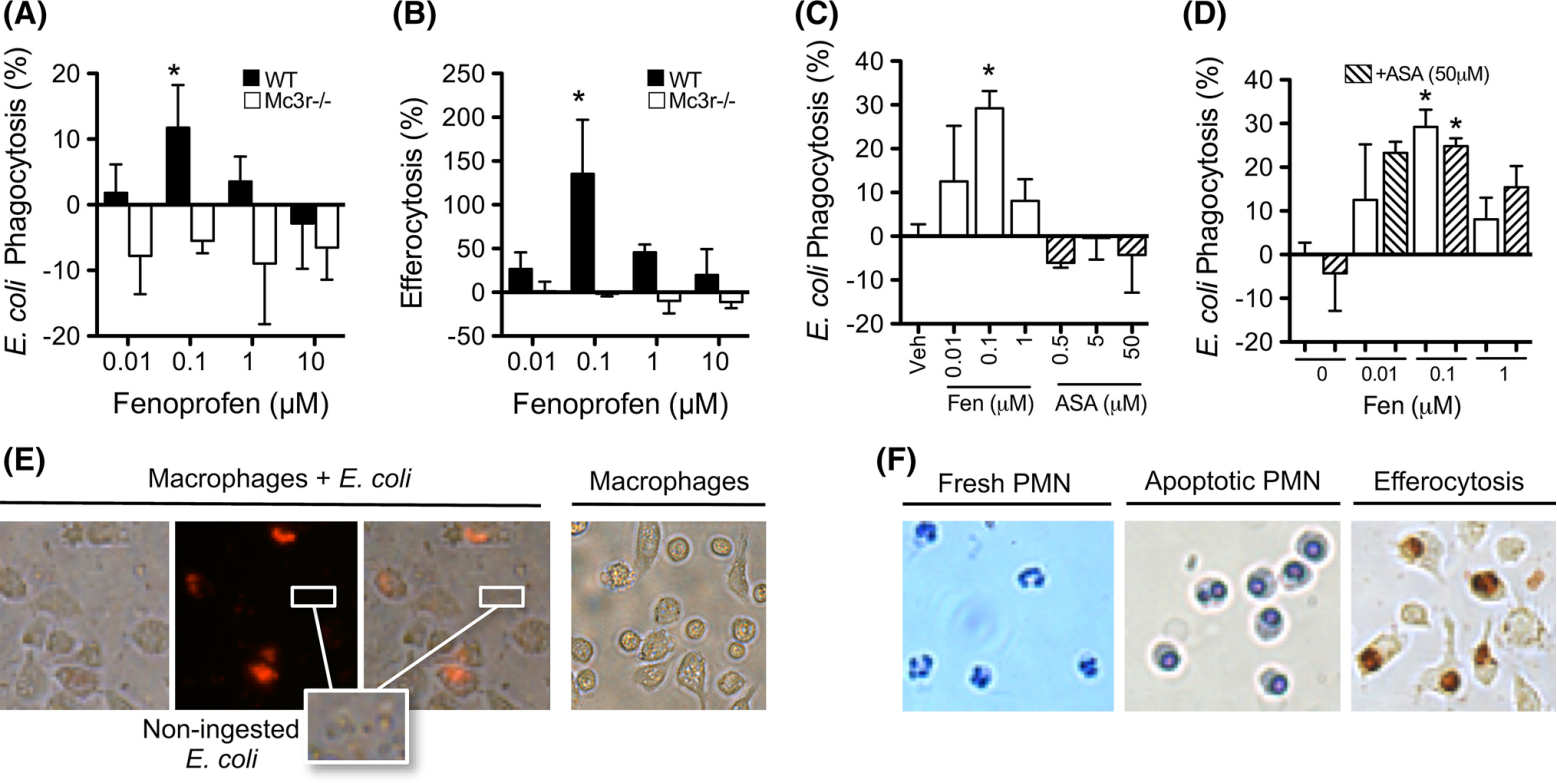
Pro-phagocytic actions of fenoprofen. Biogel-elicited peritoneal macrophages from wild type (WT) and Mc3r−/− mice were incubated with: **a** pHrodo™ Red *E. coli* for 20 min and analyzed by flow cytometry, or **b** apoptotic human neutrophils for 1 h and analyzed by myeloperoxidase (MPO) staining and cell counting. **c** Fenoprofen (Fen) and acetylsalicylic acid (ASA) were added 30 min prior addition of *E. coli.* **d** ASA was added 20 min before fenoprofen, and *E. coli* was added after 30 min incubation with fenoprofen. *Images* in **e** show that fluorescence develops specifically on ingested bacteria. *Images* in **f** show neutrophils (*dark brown* MPO staining) ingested by macrophages (efferocytosis). Data are mean ± SEM of *n* = 3; *p < 0.05 ANOVA followed by Bonferroni multiple comparison test

We next studied if fenoprofen could produce known MC_3_-mediated effects, such as increased phagocytosis by murine peritoneal macrophages [30]. The latter cell type provides a good model system for studying the effects of MC_3_ PAMs since these cells produce endogenous peptides (Fig. 4b) and express the melanocortin receptors MC_1,3,5_ [30]. As reported in Fig. 4, fenoprofen promoted phagocytosis of bacteria (Fig. 5a, e) and apoptotic neutrophils (Fig. 5b, f), the latter process often termed efferocytosis. The potentiation of phagocytosis by fenoprofen was marked and followed a bell-shaped curve, commonly observed for melanocortin activation [13, 14], with 0.1 μM being the most active concentration. Of interest fenoprofen was inactive when Mc3r−/− cells were used (Fig. 5a, b), although Mc3r−/− cells in vitro produced similar levels of endogenous agonists (Fig. 4b).

To exclude COX inhibition as part of the pro-phagocytic actions of fenoprofen, aspirin (ASA) was chosen as a control for the next study as it inhibits COX irreversibly and it is chemically unrelated to fenoprofen. While the pro-phagocytic and pro-efferocytic effect of fenoprofen was confirmed, ASA did not have any effect on phagocytosis (Fig. 5c). In a subsequent set of experiments, the pro-phagocytic effects of fenoprofen were assayed 20 min after pre-incubation with ASA (50 μM) to irreversibly block COX enzymes. As shown in Fig. 5d, the pro-phagocytic effect of fenoprofen was retained even in experimental settings with cells pre-treated with ASA.

These results, together with the experiment conducted with Mc3r−/− cells (Fig. 5a, b), indicate that this receptor genuinely mediates specific pro-resolving actions of fenoprofen.

## Discussion

The so-called Eroom’s law [38] refers to the decline in pharmaceutical R&D efficiency despite marked improvements in technology, where the cost of developing a new drug roughly doubles every 9 years. However, some estimates suggests the cost of repositioning a drug may drop from $2–3 billion to $300 million, leading to an increase in new companies entirely focused on this strategy [31]. Drug repositioning is proven attainable as several drugs have already been repurposed—e.g., the antihypertensive minoxidil for hair loss, or the antimalarial hydroxychloroquine for arthritis—although in many cases these novel indications are discovered by serendipity. However, the advance strategy resides in incorporating repositioning as an active systematic approach to drug discovery, for instance, by including known drugs in compound screening programmes.

Targeting the melanocortin system is emerging as a potential therapeutic approach to treat a variety of inflammatory conditions. This approach is corroborated not only by the large amount of scientific literature [5, 15, 32, 40], but also by the clinical evidence obtained from the use of ACTH in clinical practice since its approval in 1952. This natural melanocortin peptide has been used to treat gouty arthritis for more than 60 years and it is currently included in the ACR guidelines for the treatment of acute attacks of this disease of the joints [22, 36]. Acthar^®^ Gel and Synacthen Depot^®^ are the formulations currently available and conditions such as RA, multiple sclerosis, inflammatory bowel disease, and dermatitis fall within their FDA- and EMA-approved indications [26]. With the exception of gouty arthritis and/or specific regions using clinical evidence-based practice, ACTH is otherwise seldom used. The main reason for its limited use in patients is related to the persistent activation of MC_2_, which leads to the excessive production of cortisol with the instauration, over time, of substantial side effects.

However, the development of new MC drugs as an anti-inflammatory strategy is experiencing a renewed interest due to the discovery of MC_3_ activation as a crucial pathway mediating the anti-arthritic actions of ACTH. This “ACTH renaissance” applies to conditions like multiple sclerosis [2, 37], proteinuric nephropathies [16], and lupus [24], where the anti-inflammatory properties of ACTH might also be mediated through the peripheral, or non-adrenal, MC system. On the other hand, lack of receptor selectivity achieved by currently developed MC molecules may limit their clinical development.

While challenging, the complexity of GPCR biology represents a source of innovation in drug discovery. For example, we recently characterized a small molecule that activated only a subset of signaling cascades associated with MC_3_ (a process known as GPCR-biased signaling) with the functional consequences of retaining full anti-inflammatory effect while preventing the unwanted melanogenic effects [28]. In the present work, we exploit the existence of GPCR-allosterism as an opportunity to achieve selectivity among highly conserved receptor families, as it is the case within the MC system. Two reports have already identified positive and negative allosteric modulators to MC_4_ and MC_5_, respectively [18, 33]. In more general terms, the utility of PAMs as a therapeutic strategy in the clinic is exemplified by diazepam (Valium^®^), which potentiates the activity of GABA.

In our study, we performed a compound screening on a commercially available library aiming to identify novel agonists and/or PAMs of the human MC_3_. In this way, we integrated a drug repositioning approach (using a library containing known drugs) with the exploitation of novel capabilities of GPCRs like allosteric enhancing. Intriguingly, the anti-inflammatory drug fenoprofen, a COX inhibitor with high efficacy in RA, was identified as a potent hit. This PAM activity was, however, not specific for MC_3_, as the potency of αMSH, ACTH, and Lys-γ_3_MSH in MC_4_ and MC_5_ transfected cells was also increased. Although fenoprofen did not display receptor selectivity, as a PAMs it could offer a second wave of functional selectivity by acting only when endogenous ligands are expressed. From our perspective, the novel aspect of our findings resides in the fact that a drug with indication for RA and OA might be engaging the melanocortin system, providing proof-of-concept for MC_3_ targeting as a treatment of joint diseases.

A growing body of evidence supports the role of melanocortins and MC_3_ in joint inflammation. Melanocortin drugs can reduce cytokine levels, prevent immune cell infiltration, reduce joint inflammation, modulate nitric oxide production, and promote pro-resolving actions like efferocytosis [11, 30, 34, 41]. Many of these parameters were also affected by fenoprofen when tested in experimental arthritis. The target cells of MC agonists, relevant in the context of inflammation, are immune cells such as macrophages [4] and neutrophils [6] as well as non-immune cells in the joint. For example, MC_3_ is particularly important for osteoclast biology, as these cells show increased resorptive activity when derived from Mc3r−/− macrophages compared to WT cells [34]. Osteoblasts can also be targeted by melanocortins as shown for ACTH [43], while chondrocyte anabolic activity can be stimulated by MC agonists [20, 21], counteracting the detrimental effects of pro-inflammatory cytokines.

We provide evidence for this fundamental role of MC_3_ with further in vivo and in vitro experiments. In the model of K/BxN serum transfer arthritis [10], at 10 mg/kg daily, fenoprofen reduced clinical score by 80% in WT mice, while this effect was markedly attenuated in animals lacking MC_3_. Under these conditions, fenoprofen reduced disease incidence to 60% compared to 100% in vehicle-treated mice, an effect only observed in WT mice. These results suggest an important contribution of the receptor MC_3_ in the anti-arthritic actions of fenoprofen. We note here that, among the propionic acid COX inhibitors, fenoprofen is the only one associated with body weight loss, at least upon prolonged administration [17]. We reason this effect can be secondary to the MC_4_ activation we describe here: this compound—likely to cross the blood brain barrier—might activate MC_4_ in multiple brain regions to reduce appetite [3]. Another adverse reaction reported for fenoprofen is increased sweating (~5% incidence), which would be consistent with MC_5_ activation on exocrine glands [19].

Presence of endogenous MC peptides in the arthritic joints of K/BxN serum-treated mice is remarkable since fenoprofen allosteric enhancing activity would manifest only in their presence. At peak of arthritis, it was important to quantify a selective increase in ACTH and αMSH levels in the joints, with no concomitant changes in blood levels. Of note, it has been documented that endogenous MC peptides levels are increased in human synovial fluid from RA patients [7].

It was then crucial to compare the contribution of COX inhibition to the PAM activity of fenoprofen; hence, we tested the ability of a panel of propionic acid derivative NSAIDs to left-shift the cAMP concentration response curve. None of the drugs tested, apart from fenoprofen and its calcium salt, displayed PAM activity. This suggests on one hand, the existence of specific structural and conformational requirements for a small molecule MC_3_ PAM and, on the other hand, no associated requirement for COX inhibition to achieve this specific enhancing property.

In the final part of the study, we determined if fenoprofen could evoke MC_3_-dependent pro-resolving functions [35, 39], properties described for the melanocortin pan-agonist AP214 [30]. The pro-phagocytic and pro-efferocytic actions of fenoprofen were absent when tested with macrophages from Mc3r−/− mice. In these controlled settings aspirin and ibuprofen were inactive providing strong indication that these biological effects are unrelated to COX inhibition.

An important control that could not be run was the assessment of the contribution of COX inhibition to the anti-arthritic effects of fenoprofen because in the K/BxN serum-induced model of arthritis, mice lacking the COX-1 isoform do not develop disease [8], whereas selective COX-2 inhibitors are inactive. However, the in vitro phagocytosis assays allowed us to address this issue, since aspirin was ineffective on its own and did not affect the pro-phagocytic properties of fenoprofen.

In conclusion, MC receptors represent novel targets for the development of innovative therapies for RA and other inflammatory diseases: targeting MC_3_ using PAMs constitutes a viable and biologically effective means to reduce synovial inflammation. This work also provides the first link between MC_3_ targeting and anti-arthritic efficacy in man: fenoprofen, in contrast to other propionic acid derivatives, is particularly effective in RA and OA and here we show that activity at MC receptors, only achieved by this molecule, might explain this therapeutic advantage over other NSAIDs. Our approach also reflects the potential of repositioning screening strategies, to rediscover new mechanisms and actions for old drugs. These results may pave the way for further identification and development of new MC_3_ allosteric enhancers, and possibly also for MC_1_ and/or MC_5_, as discussed above, to modulate host inflammatory responses in chronic pathologies.

## Materials and methods

### Compound library screening

The compound screening was performed on the commercially available library NINDS (National Institute of Neurological Disorders and Stroke) containing >1400 drugs using a single concentration of test compound (10 μM). The HTRF^®^ cAMP assay (Cisbio Bioassays, Codolet, France) was performed on CHO cells stably expressing the human MC_3_ (GeneBLAzer^®^ beta-lactamase Reporter Technology, Invitrogen, Paisley, UK) according to manufacturer’s protocol. Human MC_1_, MC_4_, MC_5_ (SNAP-tag Taglite^®^ Technology, HEK293 cells; Cisbio Bioassays), human MC_2_ (GeneBLAzer^®^, CHO cells) and mouse MC_3_ (FLAG-tag, HEK293 cells; Genecopoeia, Source BioScience, Nottingham, UK) were used to study selectivity and ortholog activation. Drugs were tested for agonistic activity, in which the HTRF cAMP accumulation assay was performed with 10-point 0.5 log serial dilutions (0.316–10 μM) and for their ability to potentiate the effect of the agonist Lys-γ_3_MSH (Cambridge Biosciences, Cambridge, UK) used at EC_20_. Leftwad-shifts assays were then conducted on positive hits using a concentration response curve of Lys-γ_3_MSH (0.01–1 μM) in the absence or presence (0.1–100 μM) of test compound.

### Animals

7–8 weeks old, male, C57BL/6J wild-type (WT) mice were purchased from Charles River (Kent, UK). Mc3r−/− mice were a generous gift of Dr Chen (Merck Laboratories). All animal studies were approved and performed under the guidelines of the Ethical Committee for the Use of Animals, Barts and The London School of Medicine and Home Office Regulations (Guidance on the Operation of Animals, Scientific Procedures Act, 1986).

### Zymosan-induced peritonitis

Peritonitis was induced by the injection of 1 mg zymosan A (Sigma-Aldrich, Poole, UK) i.p. in 0.5 ml sterile PBS. Animals (4 per group) were pre-treated with fenoprofen or vehicle (PBS) administered i.p. 30 min before zymosan injection. Four hours later, mice were killed by CO_2_ exposure and peritoneal cavities washed with 4 ml of ice cold PBS containing 3 mM EDTA and 25U/ml heparin. 100 µl aliquots of lavage fluids were stained with Turk’s solution (0.01% crystal violet in 3% acetic acid) and cells counted on a Neubauer haemocytometer or stained with conjugated antibodies for Ly-6G (FITC) and F4/80 (APC) used in 1:100 dilution. Corresponding isotype controls and blocking antibody anti-mouse CD16/32 were used. All antibodies were purchased from eBioscience (Hatfield, UK). Flow cytometry was performed in the BD FACSCalibur™.

### K/BxN serum transfer arthritis model

Arthritis was induced with two i.p. injections of 100 μl of K/BxN serum on days 0 and 2. Disease was monitored daily until day 8 by assessing the paw volume using a plethysmometer (Ugo Basile, Comerio, Italy), disease incidence (mice showing any signs of arthritis) and clinical score (score per paw: 0 = no signs of inflammation; 1 = subtle inflammation, localized; 2 = easily identified inflammation but localized; 3 = evident inflammation, not localized; max score = 12 per mouse) [23]. Pharmacological treatments were administered i.p. twice daily from day 2 until the end of the experiment. Fenoprofen calcium salt hydrate and ibuprofen sodium salt were obtained from Sigma-Aldrich (Poole, UK).

### RNA extraction, cDNA synthesis, and real time-PCR

Total RNA was extracted using TRIzol (Invitrogen, Paisley, UK) following manufacturer’s instructions. cDNA was synthesized using 1ug of RNA with the SuperScript III Reverse Transcriptase (Invitrogen, Paisley, UK). Real time-PCR was performed in duplicates, with 2 μl cDNA diluted 1/5, 1 μl primers and Power SYBR Green PCR Master Mix (Applied Biosystems, Warrington, UK), using the ABI Prism 7900HT Sequence Detection System. Quantitect primers (QIAGEN, Crawley, UK) used are the following: Hprt (QT00166768), Cd14 (QT00246190), Il1b (QT01048355), Tnfsf11 (QT00147385), Tnfrsf11b (QT00106757), Ctsk (QT00150703) and Mmp9 (QT00108815). Dissociation step was always included to confirm the absence of un-specific products. Fold change was calculated as 2^−ΔΔCt^ using Hprt as reference gene.

### Enzyme immunoassay

Melanocortin peptide levels were determined by enzyme immunoassay (EIA) in plasma and proteins extracts prepared from the right upper limbs homogenized in RIPA buffer using the Precellys^®^24 tissue homogenizer (Stretton Scientific, Derbyshire, UK) and hard tissue grinding beads. Alpha-MSH, gamma2-MSH, and ATCH EIA kits (Phoenix Pharmaceuticals, Karlsruhe, Germany) were used following manufacturers protocols.

### Histological analyses

Joints were fixed with 4% neutral buffered formalin, decalcified with 10% formic acid and paraffin embedded. Sections (4 μm) were stained with hematoxylin and eosin (H&E) and fast green and safranin-O. Sections were graded from 0 (no disease) to 3 (severe) based on the degree of synovitis and cartilage erosion [34].

### *Escherichia coli* phagocytosis by macrophages

Biogel-elicited peritoneal macrophages were obtained from wild type and Mc3r−/− mice 4 days after an i.p. injection with 1 ml of 2% biogel (BioRad, Hemel Hempstead, UK) [27]. 1 × 10^6^ cells were plated in 24-well plates and pre-treated with corresponding drugs for 30 min before addition of bacteria (pHrodo™ Red *E. coli* Bioparticles^®^ Conjugate, Invitrogen, Paisley, UK). After 20 min, non-ingested bacteria were washed and cells incubated for further 30 min to allow fluorescence to develop. Cells were trypsinized and analyzed by flow cytometry (BD FACSCalibur™, FL-2).

### Phagocytosis of apoptotic neutrophils by macrophages

Experiments using healthy volunteers were approved by the local research ethics committee (P/00/029 East London and The City Local Research Ethics Committee 1). Informed written consent was provided according to the Declaration of Helsinki. Neutrophils were isolated from human blood using the double-density gradient with Histopaque 10771 and 11191 following manufacturer’s protocols [27]. Cells were incubated overnight in 10% FCS to let neutrophils undergo spontaneous apoptosis. 0.5 × 10^6^ biogel-elicited macrophages were plated in 24-well plates and pre-treated with corresponding drugs for 30 min before addition of apoptotic neutrophils. After 1 h, macrophages were washed and fixed with 2.5% glutaraldehyde. The myeloperoxidase (MPO) assay was performed by adding 0.1 mg/ml of *o*-dianisidine dihydrochloride (Sigma-Aldrich, Poole, UK) and 0.03% (v/v) hydrogen peroxide. After 1 h, cells were washed and analyzed by light microscopy, with three random fields being acquired per well.

### Statistics

Data are reported as mean ± SEM of n animals or tissue samples or, for in vitro experiments, performed in duplicate or triplicate from at least three distinct experiments. A *p* value <0.05 was taken as significant.

### Electronic supplementary material

Below is the link to the electronic supplementary material.

Supplementary material 1 (TIFF 3790 kb) **Supplementary Figure S1. Agonistic activity of fenoprofen.** cAMP production upon melanocortin receptor activation was studied in MC_1_, MC_4_ and MC_5_ transfected HEK293 cells (SNAP-tag Taglite^®^ Technology, Cisbio bioassays, Codolet, France) and MC_2_ transfected CHO-K1 cells (GeneBLAzer^®^ beta-lactamase Reporter Technology, Invitrogen, Paisley, UK). Fenoprofen was tested to explore its potential agonistic activity against MC_1_ (A), MC_2_ (B), MC_4_ (C) or MC_5_ (D). Control cells (transfected with empty vector DNA) were used to confirm lack of activity of NDP-αMSH and fenoprofen in HEK293 cells in the absence of melanocortin receptors (E). The effect on MC_1_ could not be accurately addressed due to the known high constitutive activity of this receptor

Supplementary material 2 (TIFF 1584 kb) **Supplementary Figure S2.** Ibuprofen does not modulate human and mouse MC_3_ receptor. cAMP production upon ibuprofen treatment was studied in: (A) human MC_3_ transfected CHO-K1 cells (GeneBLAzer^®^ beta-lactamase Reporter Technology, Invitrogen, Paisley, UK) and (B) mouse MC3 transfected HEK293 cells (FLAG-tag, Genecopeia, Source BioScience, Nottingham, UK) in the presence of αMSH

Supplementary material 3 (TIFF 3102 kb) **Supplementary Figure S3.** Dose response effect of fenoprofen in the zymosan-induced peritonitis model. Peritonitis was induced with 1 mg zymosan injected i.p., 30 min after drug administration. Mice (C57BL/6) were sacrificed 4 h later and peritoneal cells analyzed by cell counting and flow cytometry. (A) Total cells per mouse; (B) neutrophils (Ly6G^hi^/F4/80-); (C) inflammatory monocytes (Ly6G^low^/F4/80 +); (D) effective dose (ED_50_) calculated on neutrophil counts. Data are mean ± SEM of n = 5; *p < 0.05 ANOVA followed by Bonferroni multiple comparison test

Supplementary material 4 (TIFF 9798 kb) **Supplementary Figure S4.** Histologial scoring criteria using H&E and safranin-O staining. Tissue Sects. (4 µm) were stained with hematoxylin and eosin (H&E) and fast green and safranin-O. Sections were graded from 0 (no disease) to 3 (severe) based on the degree of synovitis (purple staining in the H&E sections, C) and cartilage erosion (loss of red coloration in the safranin-O sections)

Supplementary material 5 (TIFF 974 kb) **Supplementary Figure S5.** Plasma levels of endogenous melanocortin peptides. αMSH (A), γMSH (B) and ACTH (C) were determined by EIA in plasma collected at day 8 from arthritic (K/BxN) and control mice. Data are mean ± SEM of n = 5, analyzed by *t* test (no significant changes were detected)

Supplementary material 6 (DOCX 55 kb)
