# Supplementary figures and images for: Old drugs with new skills: fenoprofen as an allosteric enhancer at melanocortin receptor 3
Source: Cell Mol Life Sci. 2016 Nov 16;74(7):1335–45. doi: 10.1007/s00018-016-2419-3 (PMC5346439; doi:10.1007/s00018-016-2419-3)

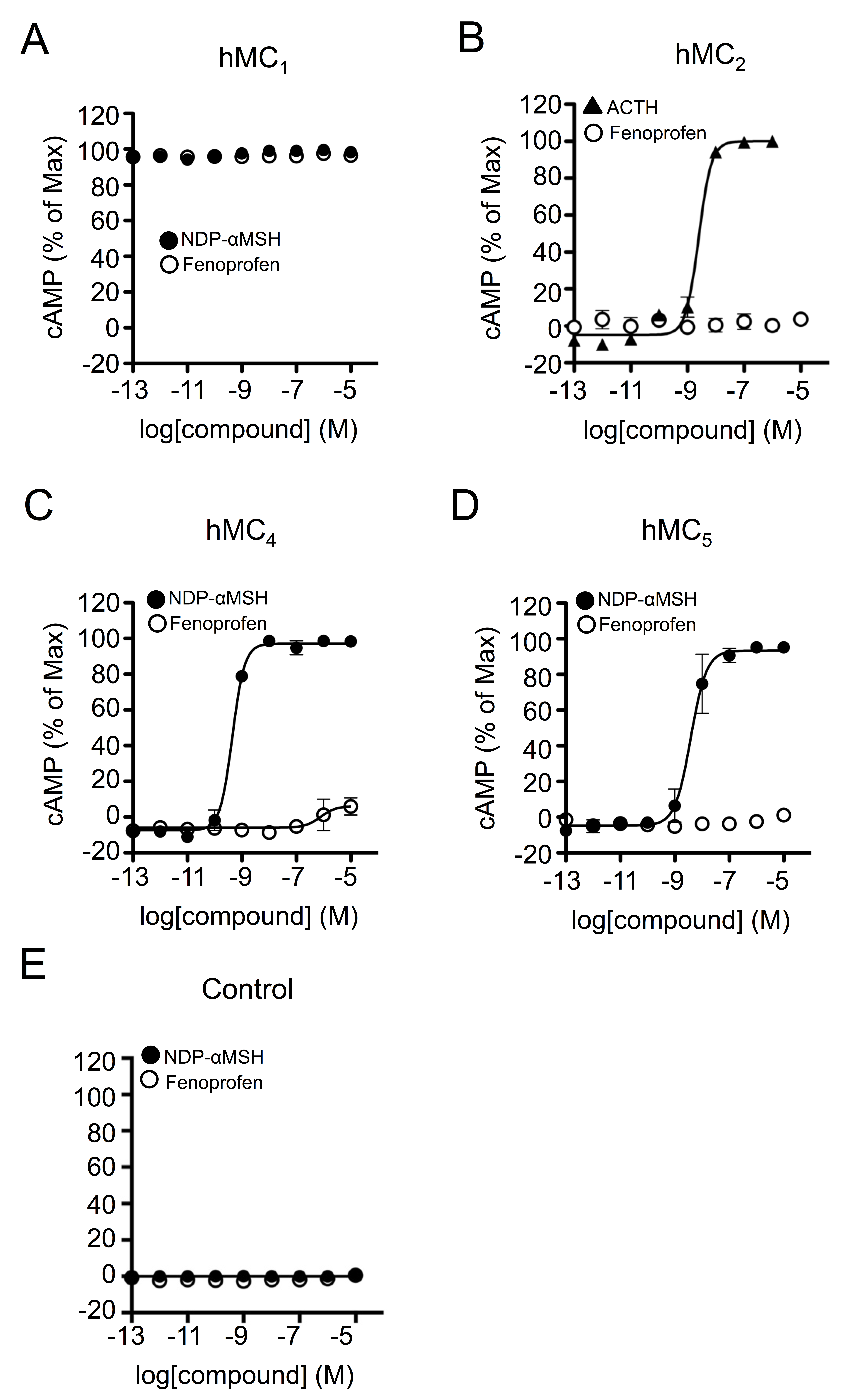

Supplement: Supplementary file 1 — Supplementary material 1 (TIFF 3790 kb) Supplementary Figure S1. Agonistic activity of fenoprofen. cAMP production upon melanocortin receptor activation was studied in MC1, MC4 and MC5 transfected HEK293 cells (SNAP-tag Taglite® Technology, Cisbio bioassays, Codolet, France) and MC2 transfected CHO-K1 cells (GeneBLAzer® beta-lactamase Reporter Technology, Invitrogen, Paisley, UK). Fenoprofen was tested to explore its potential agonistic activity against MC1 (A), MC2 (B), MC4 (C) or MC5 (D). Control cells (transfected with empty vector DNA) were used to confirm lack of activity of NDP-αMSH and fenoprofen in HEK293 cells in the absence of melanocortin receptors (E). The effect on MC1 could not be accurately addressed due to the known high constitutive activity of this receptor [file 18_2016_2419_MOESM1_ESM.tiff]

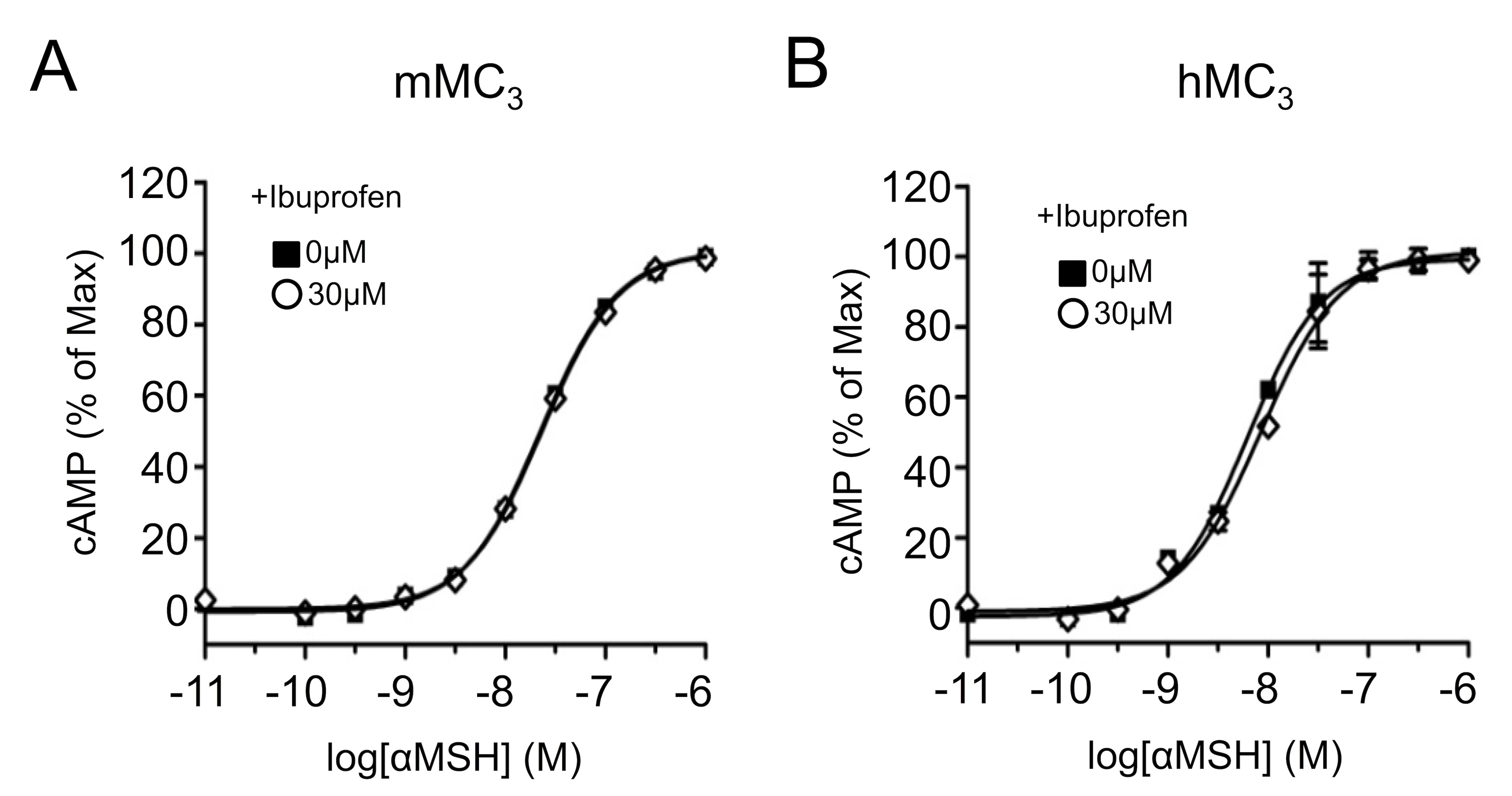

Supplement: Supplementary file 2 — Supplementary material 2 (TIFF 1584 kb) Supplementary Figure S2. Ibuprofen does not modulate human and mouse MC3 receptor. cAMP production upon ibuprofen treatment was studied in: (A) human MC3 transfected CHO-K1 cells (GeneBLAzer® beta-lactamase Reporter Technology, Invitrogen, Paisley, UK) and (B) mouse MC3 transfected HEK293 cells (FLAG-tag, Genecopeia, Source BioScience, Nottingham, UK) in the presence of αMSH [file 18_2016_2419_MOESM2_ESM.tiff]

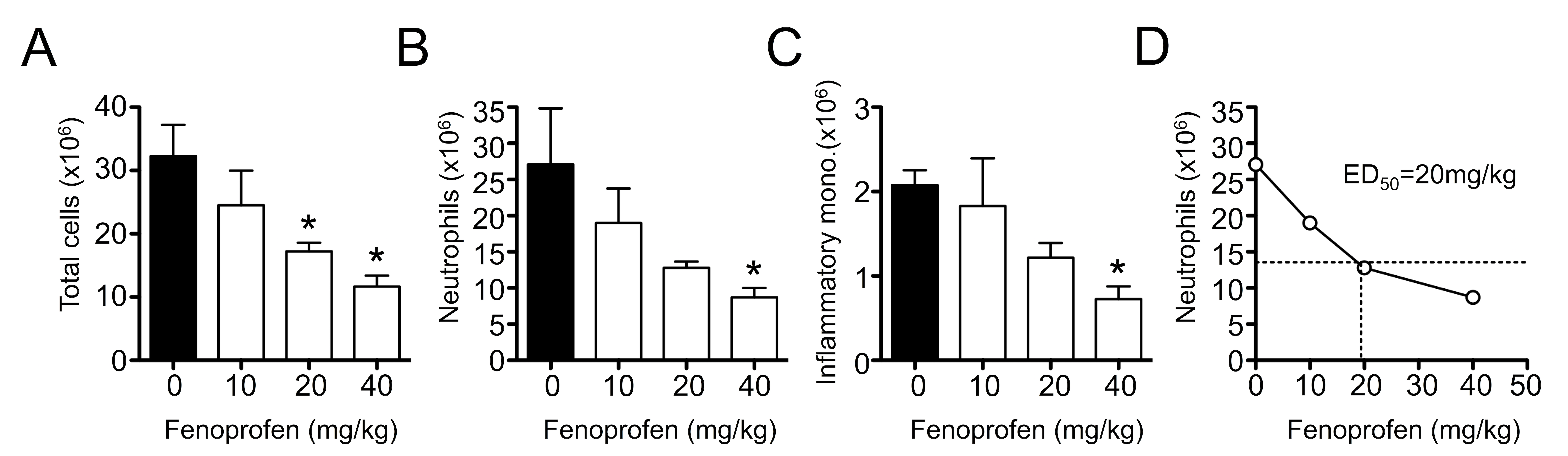

Supplement: Supplementary file 3 — Supplementary material 3 (TIFF 3102 kb) Supplementary Figure S3. Dose response effect of fenoprofen in the zymosan-induced peritonitis model. Peritonitis was induced with 1 mg zymosan injected i.p., 30 min after drug administration. Mice (C57BL/6) were sacrificed 4 h later and peritoneal cells analyzed by cell counting and flow cytometry. (A) Total cells per mouse; (B) neutrophils (Ly6Ghi/F4/80-); (C) inflammatory monocytes (Ly6Glow/F4/80 +); (D) effective dose (ED50) calculated on neutrophil counts. Data are mean ± SEM of n = 5; *p < 0.05 ANOVA followed by Bonferroni multiple comparison test [file 18_2016_2419_MOESM3_ESM.tiff]

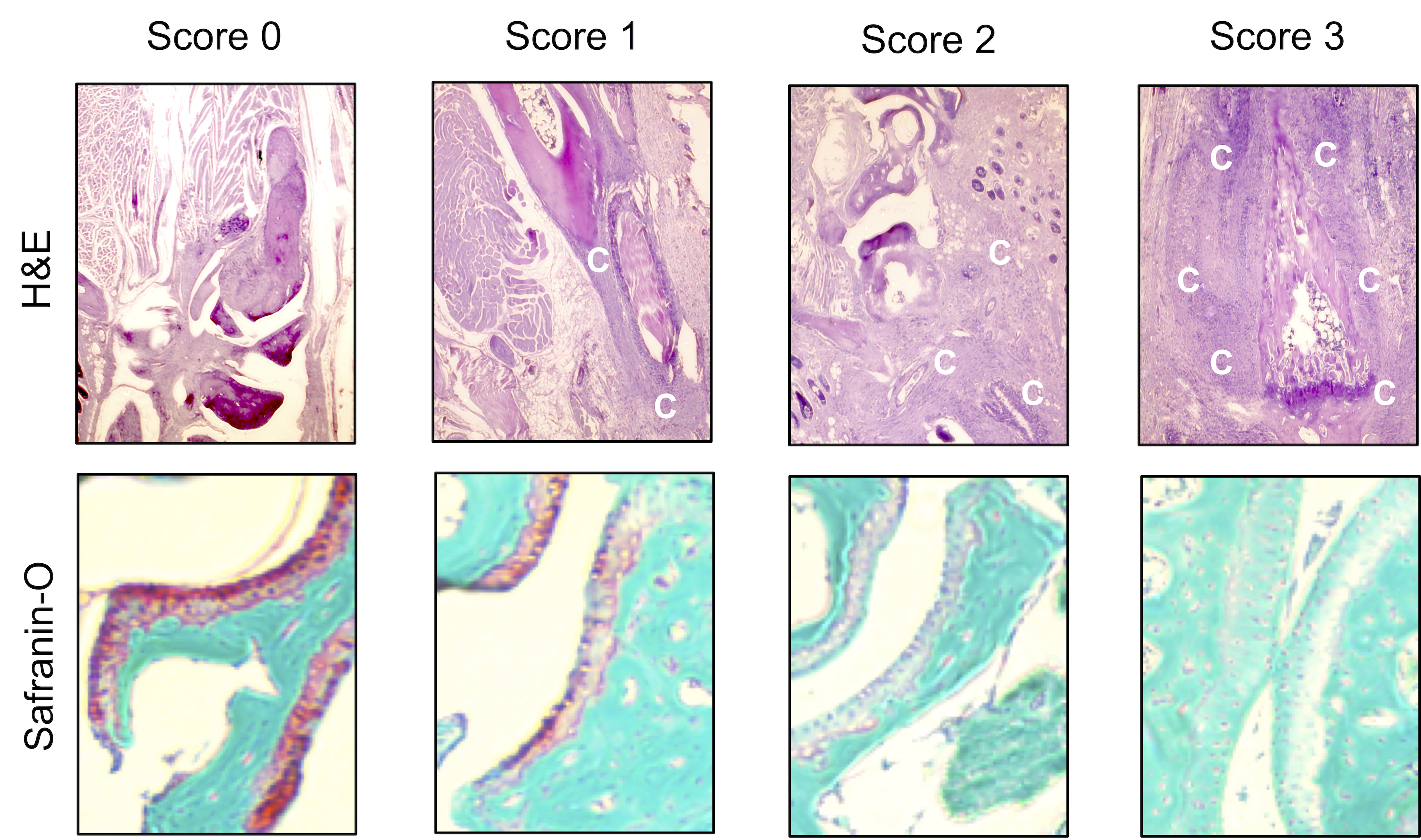

Supplement: Supplementary file 4 — Supplementary material 4 (TIFF 9798 kb) Supplementary Figure S4. Histologial scoring criteria using H&E and safranin-O staining. Tissue Sects. (4 µm) were stained with hematoxylin and eosin (H&E) and fast green and safranin-O. Sections were graded from 0 (no disease) to 3 (severe) based on the degree of synovitis (purple staining in the H&E sections, C) and cartilage erosion (loss of red coloration in the safranin-O sections) [file 18_2016_2419_MOESM4_ESM.tiff]

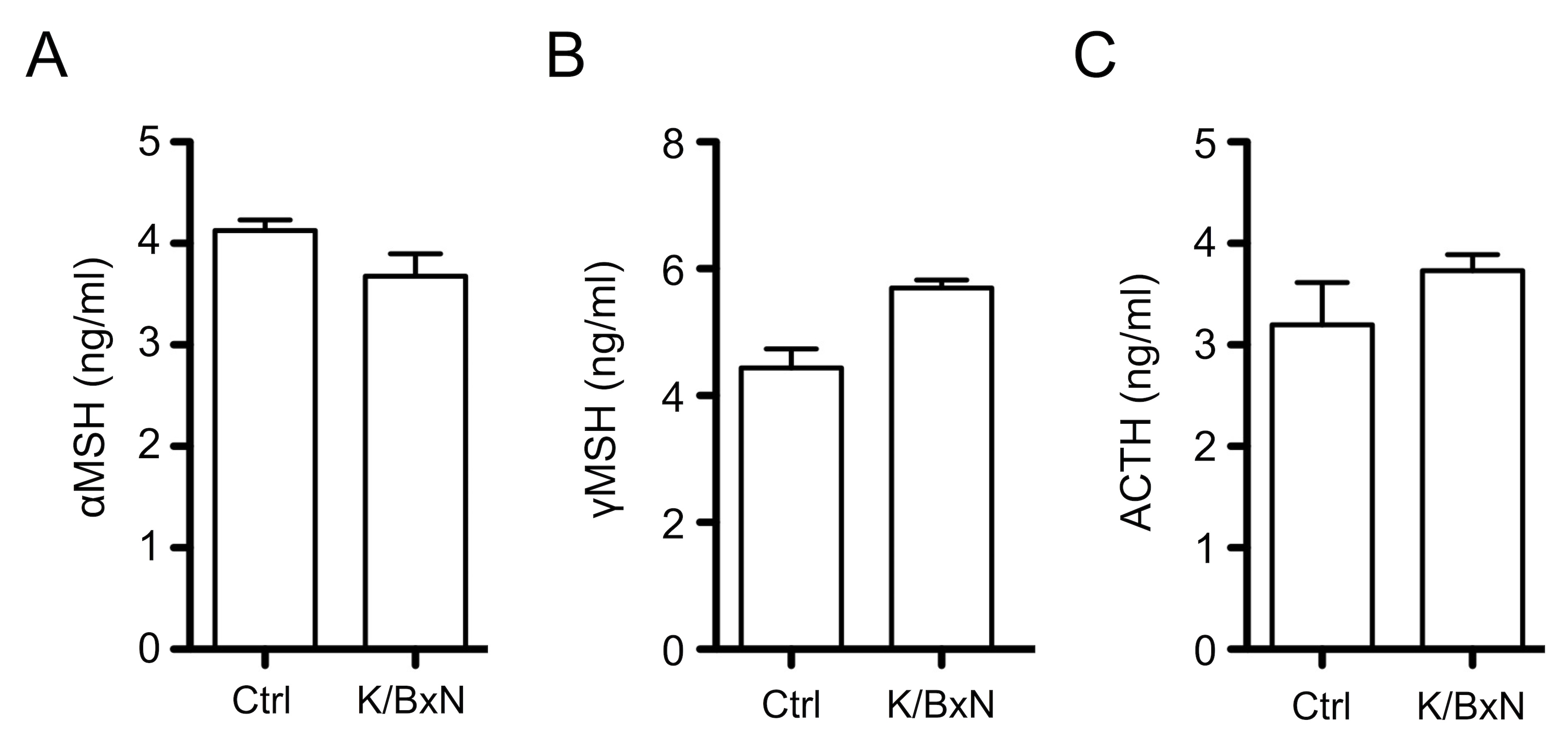

Supplement: Supplementary file 5 — Supplementary material 5 (TIFF 974 kb) Supplementary Figure S5. Plasma levels of endogenous melanocortin peptides. αMSH (A), γMSH (B) and ACTH (C) were determined by EIA in plasma collected at day 8 from arthritic (K/BxN) and control mice. Data are mean ± SEM of n = 5, analyzed by t test (no significant changes were detected) [file 18_2016_2419_MOESM5_ESM.tiff]
