# Supplementary material for: Old drugs with new skills: fenoprofen as an allosteric enhancer at melanocortin receptor 3
Source: Cell Mol Life Sci. 2016 Nov 16;74(7):1335–45. doi: 10.1007/s00018-016-2419-3 (PMC5346439; doi:10.1007/s00018-016-2419-3)
Supplement: Supplementary file 6 — Supplementary material 6 (DOCX 55 kb) [file 18_2016_2419_MOESM6_ESM.docx]

**Supplementary Table S1.** **PAM activity of COX-inhibitors of the propionic acid subgroup.** Leftward shift of the cAMP dose-response curves at MC_3_ at three different concentrations of the COX-inhibitors (*indicates PAM activity).

|  | **10μM** | | **30μM** | | **100μM** | |
| --- | --- | --- | --- | --- | --- | --- |
|  | pEC_50_ | Log shift | pEC_50_ | Log shift | pEC_50_ | Log shift |
| Naproxen | 8.86 | -0.07 | 8.83 | -0.09 | 8.76 | -0.17 |
| Ibuprofen | 8.85 | -0.07 | 8.84 | -0.08 | 8.78 | -0.14 |
| Ketoprofen | 8.87 | -0.05 | 8.83 | -0.09 | 8.71 | -0.21 |
| Flurbiprofen | 8.9 | -0.02 | 8.81 | -0.11 | 8.72 | -0.20 |
| Indoprofen | 8.86 | -0.06 | 8.86 | -0.07 | 8.79 | -0.13 |
| Suprofen | 8.94 | -0.01 | 8.89 | -0.03 | 8.83 | -0.09 |
| Carprofen | 8.92 | -0.01 | 8.93 | 0.01 | 8.77 | -0.16 |
| Fenoprofen | 9.10 | 0.18^(^*^)^ | 9.29 | 0.37^(^*^)^ | 9.46 | 0.53^(^*^)^ |
| Fenoprofen Ca | 9.12 | 0.32^(^*^)^ | 9.48 | 0.56^(^*^)^ | 9.57 | 0.64^(^*^)^ |
